# Investigation of *ATG16L1* rs2241880 Polymorphism with Cancer Risk: A Meta-Analysis

**DOI:** 10.3390/medicina55080425

**Published:** 2019-07-31

**Authors:** Abdolkarim Moazeni-Roodi, Farhad Tabasi, Saeid Ghavami, Mohammad Hashemi

**Affiliations:** 1 Department of Clinical Biochemistry, Iranshahr University of Medical Sciences, Iranshahr 9916643535, Iran; 2 Student Research Committee, Zahedan University of Medical Sciences, Zahedan 9816743463, Iran; 3 Department of Human Anatomy and Cell Science, Max Rady College of Medicine, Rady Faculty of Health Sciences, University of Manitoba, Winnipeg, MB R3E 0J9, Canada; 4 Research Institute in Oncology and Hematology, CancerCare Manitoba, University of Manitoba, Winnipeg, MB R3E 3P5, Canada; 5 Genetics of Non-communicable Disease Research Center, Zahedan University of Medical Sciences, Zahedan 9816743463, Iran; 6 Department of Clinical Biochemistry, School of Medicine, Zahedan University of Medical Sciences, Zahedan 9816743175, Iran

**Keywords:** ATG16L1, polymorphism, rs2241880, Thr300Ala, cancer, meta-analysis

## Abstract

*Background and Objectives:* Previous studies have investigated the impact of the *ATG16L1* rs2241880 (Thr300Ala) polymorphism on individual susceptibility to cancer, but the conclusions are still controversial. To get a more precise evaluation of the correlation between *ATG16L1* rs2241880 polymorphism and cancer susceptibility, we performed a meta-analysis of the association of all eligible studies. *Materials and Methods:* Searches were performed in the Web of Science, PubMed, Scopus and Google Scholar databases up to November 2018. A total of 12 case-control studies from 9 articles comprising 2254 cases and 4974 controls were included. Statistical analysis was achieved by STATA 14.1 and Review Manager 5.3 software. The odds ratios (ORs) with 95% confidence intervals (95% CIs) under five genetic models were used to determine the strength of association among rs2241880 polymorphism and cancer susceptibility. *Results:* The findings did not support an association between the rs2241880 variant in either the overall study population or the subgroups, based on cancer types and ethnicity in any of the genetic models. As far as we know, our study is the first meta-analysis of the association between rs2241880 polymorphism and cancer risk. *Conclusions:* In conclusion, the findings of this meta-analysis proposes that the *ATG16L1* rs2241880 polymorphism may not play a role in cancer development. Further well-designed studies are necessary to clarify the precise role of the *ATG16L1* rs2241880 polymorphism on cancer risk.

## 1. Introduction

Cancer is one of the main public health problem worldwide with about 18.1 million new cancer cases and 9.6 million cancer deaths in 2018 [1]. The precise mechanisms of cancer initiation and progression has remained largely unknown [2]. Mounting evidence has suggested that genetic predisposition plays a significant role in the risk of individual cancer development [3,4].

Autophagy, an evolutionarily conserved process, is important for survival, differentiation, development, and homeostasis through degrading damaged organelles and long-lived proteins [5,6,7,8]. Autophagy is a tightly regulated mechanism, regulated by several autophagy related genes (ATGs), and is classified into three subgroups, including macroautophagy (hereafter autophagy), microautophagy, and chaperon-mediated autophagy [9,10,11,12,13]. It has been documented that autophagy is involved in multiple diseases, including cancers, infectious diseases, fibrotic diseases, neurodegeneration and aging [14,15,16,17,18,19,20,21]. During cancer development, autophagy is considered a double edge sword because it can support or prevent cancer development through different mechanisms, including apoptotic cell death, chemo-resistance, tumorigenesis and metastasis [16,22,23,24,25,26].

The autophagy-related 16-like 1 gene (*ATG16L1*) is located on the long arm of chromosome 2 (2q37.1) [27]. It encodes ATG16L1, which is a component of a large protein complex essential for autophagy [28]. ATG16L1 plays an essential role in regulation of LC3 lipidation, and formation and insertion of lipidated LC3 into double membrane autophagosomes [29]. ATG16L1 is also involved in regulation of carcinogenesis in many cancers. As an example, it has been reported that the Thr300Ala variant of ATG16L1 is associated with a decrease in brain metastasis of non-small cell lung cancer [30]. The nonsynonymous rs2241880 (Thr300Ala) polymorphism in the *ATG16L1* gene is situated on coding exon 9.

Several studies that have investigated the relationship between the rs2241880 (Thr300Ala) polymorphism in *ATG16L1* and several cancers among different ethnic populations have had conflicting outcomes [31,32,33,34,35,36,37,38,39]. Therefore, for the first time, we aimed to conduct a meta-analysis of all available studies published to date to examine the impact of the *ATG16L1* rs2241880 polymorphism on cancer susceptibility.

## 2. Methods 

### 2.1. Literature Search

**Figure 1:**
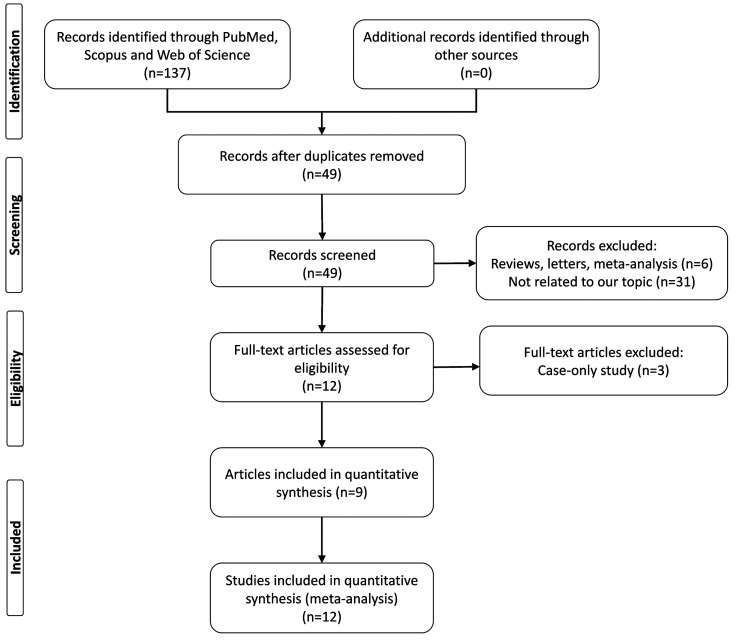
Flow chart shows the detailed study selection process of this meta-analysis.

In order to identify eligible articles, we comprehensively searched the Web of Science, PubMed, and Scopus databases, up to April 2019, for the relationship between the *ATG16L1* rs2241880 polymorphism and susceptibility to cancer. The search terms used were “ATG16L1 or autophagy related 16 like 1” and “cancer or malignant or tumor” and “polymorphism or variant or rs2241880 or T300A or +898A > G” or Thr300Ala. The selection process of eligible studies is shown in Figure 1. Studies consistent with the following criteria were included in the meta-analysis: case-control studies that focused on the correlation between the *ATG16L1* polymorphism and risk of cancer, with sufficient information for estimation of the odds ratios (ORs) and their 95% confidence intervals. Studies were excluded from consideration if not correlated to *ATG16L1* polymorphism and cancer risk; conference papers, reviews, meta-analyses; and studies without detailed genotyping data.

### 2.2. Data Extraction

Two authors screened and extracted the data from eligible studies independently. Any disagreements were discussed with the third author. The following data were extracted from each study including the first author’s name, year of publication, country, ethnicity, type of cancer, source of control, genotyping methods, sample size, as well as genotype and allelic frequencies of the cases and controls. 

### 2.3. Statistical Analysis

The Hardy–Weinberg equilibrium (HWE) of control genotypes was inspected using a χ2 test. We used pooled odds ratios (ORs) with 95% confidence intervals (CIs) to assess the strength of the association of the *ATG16L1* polymorphism with cancer risk in five genetic models. The significance of the pooled OR was determined by the *z*-test, and a *p* < 0.05 was considered statistically significant. 

Heterogeneity among the studies was assessed by using the Q statistic and the I^2^ statistic. *p* < 0.10 was considered statistically significant. The random effects model was applied if heterogeneity was observed among studies; otherwise, the fixed effects model was used.

Publication bias was inspected visually by a funnel plot and an asymmetric plot suggested a possible publication bias. Funnel plot asymmetry was measured further using the Egger and Begg tests. A *p* value < 0.05 was considered significant publication bias.

Sensitivity analysis was conducted to evaluate whether the findings were affected significantly by a single study by neglecting each study in turn to determine the effect on the pooled analysis. Statistical analyses were achieved using the STATA 14.1 software and Review Manager 5.3.

## 3. Results

### 3.1. Study Characteristics

Through the literature search and selection in accordance with the inclusion criteria, nine articles, including 12 case-control studies, comprising 2254 cases and 4974 controls, were ultimately included in the quantitative analysis (Table 1). The genotype distributions of the *ATG16L1* rs2241880 polymorphism in all subjects are shown in Table 1. The genotype distributions in the controls of the 12 studies were fitted into the HWE, except for two studies [31,35].

### 3.2. Main Analysis Results

**Figure 2:**
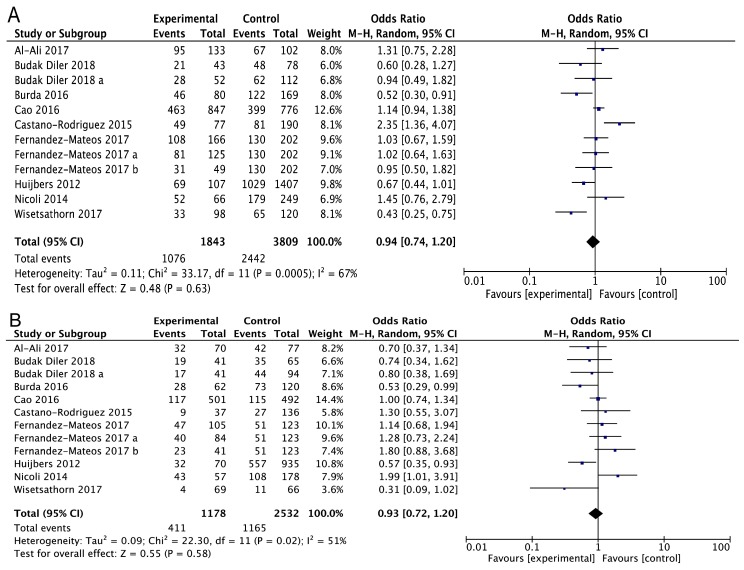

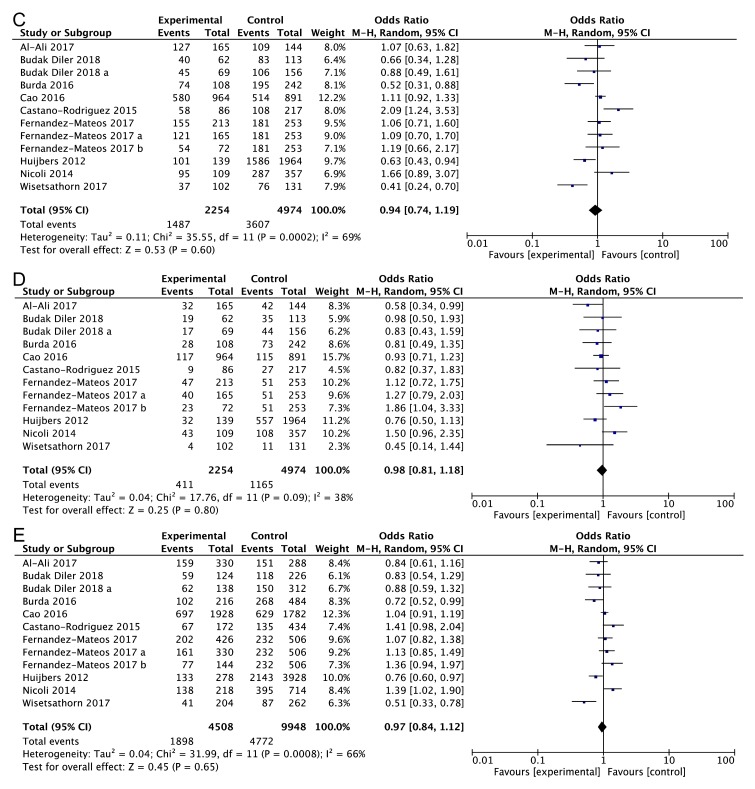
The forest plots for the association between the *ATG16L1* rs2241880 polymorphism and cancer risk for AG vs. AA (**A**), GG vs. AA (**B**), AG + GG vs. AA (**C**), GG vs. AG + AA (**D**), and G vs. A (**E**).

As shown in Figure 2 and Table 2, the findings did not support a correlation between the *ATG16L1* rs2241880 polymorphism and cancer risk. Overall, no significant associations were found for AG vs. AA (OR = 0.94, 95% CI = 0.74–1.20, *p* = 0.63, Figure 2A), CG vs. AA (OR = 0.93, 95% CI = 0.72–1.20, *p* = 0.58, Figure 2B), AG + GG vs. AA (OR = 0.94, 95% CI = 0.94–1.19, *p* = 0.60, Figure 2C), GG vs. AG + AA (OR = 0.98, 95% CI = 0.81–1.18, *p* = 0.80, Figure 2D), and G vs. A (OR = 0.97, 95%CI = 0.84–1.12, *p* = 0.65, Figure 2E). 

### 3.3. Subgroup Analysis

Stratified analysis was achieved by cancer types and ethnicity (Table 3). The stratified analysis revealed no association between the *ATG16L1* rs2241880 variant and either cancer types or ethnicities. 

### 3.4. Heterogeneity and Publication Bias

**Figure 3:**
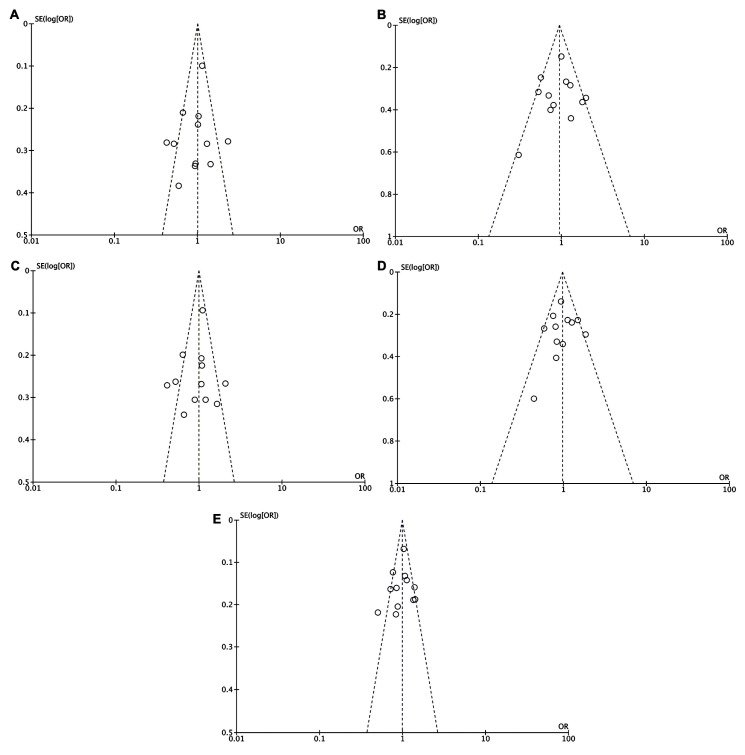
Begg’s funnel plot on publication bias for association between the *ATG16L1* rs2241880 polymorphism and cancer risk for AG vs. AA (**A**), GG vs. AA (**B**), AG + GG vs. AA (**C**), GG vs. AG + AA (**D**), and G vs. A (**E**).

There were significant heterogeneities in all genetic models examined except for the recessive model (Table 2). Begg’s funnel plot and Egger’s linear regression test revealed no apparent publication bias in our overall analysis in any genetic models (Table 2 and Figure 3). 

### 3.5. Sensitivity Analysis

**Figure 4:**
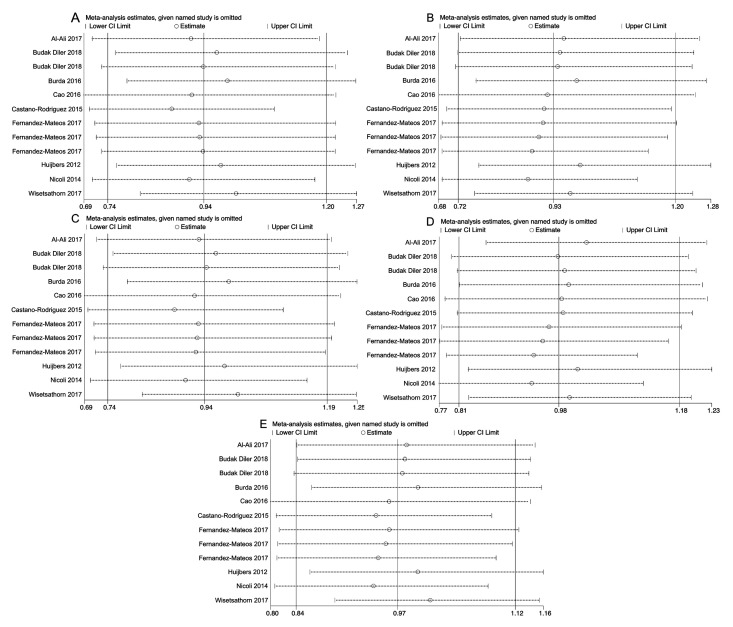
Sensitivity analyses for studies on *ATG16L1* rs2241880 polymorphism and cancer risk for AG vs. AA (**A**), GG vs. AA (**B**), AG + GG vs. AA (**C**), GG vs. AG + AA (**D**), and G vs. A (**E**).

A sensitivity analysis was done to inspect the impact of an individual study on the pooled ORs. The results indicated that the pooled ORs were not significantly affected by a single study, suggesting that the pooled results are reliable (Figure 4).

## 4. Discussion

It has been shown that the nonsynonymous rs2241880 (Thr300Ala) polymorphism of the *ATG16L1* gene affects the autophagy process [40] and also modulates the production of interleukin-1 beta (IL-1β) in human cells [41]. The exact effect of the *ATG16L1* rs2241880 polymorphism on the pathogenesis of cancer is not fully understood. Several studies investigated the impact of the *ATG16L1* rs2241880 polymorphism on susceptibility to cancer. Al-Ali et al. [39] reported that the rs2241880 variant significantly decreased the risk of lung cancer in a Spanish population. Budak Diler et al. [31] showed that the rs2241880 variant was not associated with the risk of prostate cancer or bladder cancer in a Turkish population. Burada et al. [32] found that the rs2241880 polymorphism was associated with protection against gastric cancer in a Romanian population. Cao et al. [38] found no significant association between the rs2241880 variant and colorectal cancer in a Chinese population. Castano-Rodriguez [33] reported that the rs2241880 polymorphism significantly increased the risk of gastric cancer in a Singaporean population. Fernandez-Mateos et al. [34] showed that the rs2241880 variant significantly increased the risk of oral cavity cancer but the variant was not associated with the risk of laryngeal cancer or pharyneal cancer in a Spanish population. Huijbers et al. [35] revealed that the rs2241880 variant was associated with protection against thyroid cancer in a Netherlander population. Nicoli et al. [36] showed that rs2241880 variant significantly increased the risk of colorectal cancer in a Romanian population. Wisetsathorn et al. [37] observed that the rs2241880 variant significantly increased the risk of hepatocellular carcinoma in a Thai population. Figlioli et al. [42] proposed that the *ATG16L1* rs2241880 variant significantly decreased the risk of thyroid cancer. Due to insufficient data this study was excluded from the meta-analysis.

To the best of our knowledge, this is the first meta-analysis that aimed to investigate the possible association between the *ATG16L1* rs2241880 gene polymorphism and overall cancer susceptibility. Our findings showed no significant association between the rs2241880 polymorphism of the *ATG16L1* gene and cancer susceptibility in any genetic models. The results of this meta-analysis are not consistent with some previous studies [32,33,34,36,37]. The discrepancy between studies may be attributed to small sample sizes, type of cancer and different genetic backgrounds among the diverse ethnicities of the above-mentioned studies.

How the rs2241880 (Thr300Ala) polymorphism alters the biology of ATG16L1 is not yet known. Yuan et al. [43] showed that the *ATG16L1* rs2241880 polymorphism was significantly associated with survival in lung adenocarcinoma patients.

In spite of the heterogeneity across studies, no evidence of publication bias was detected by either Begg’s or Egger’s tests. In addition, the sensitivity analysis did not significantly alter the overall results for all genetic models, which implies stability and reliability for our findings. 

This meta-analysis has some limitations that should be taken into account. First, only published articles in English were included in the pooled analysis because data in other languages and data from other ongoing studies were not available. Second, heterogeneity was observed among the studies, which have distorted the conclusion. The heterogeneity among studies may be due to differences in cancer types and ethnicities. Third, we calculated crude ORs, which were unadjusted estimations. Fourth, due to the lack of raw data, we were unable to perform gene–environment interactions. Finally, the number of individual studies for each cancer type was inadequate for stratified analysis. Our findings should therefore be interpreted with caution.

## 5. Conclusions

In conclusion, the current study is the first meta-analysis to evaluate the association between the *ATG16L1* rs2241880 polymorphism and the risk of cancer. Our results did not support an association between the *ATG16L1* rs2241880 polymorphism and cancer risk. Larger well-designed studies are needed to elucidate the exact role of the *ATG16L1* rs2241880 polymorphism on cancer risk.

## Figures and Tables

**Table 1:** Characteristics of all studies included in the meta-analysis.

First Author	Year	Country	Ethnicity	Cancer Type	Source of Control	Genotyping Method	Case/Control	Cases					Controls					HWE (P)
								AA	AG	GG	A	G	AA	AG	GG	A	G	
Al-Ali et al. [39]	2017	Spain	Caucasian	Lung cancer	PB	TaqMan	165/144	38	95	32	171	159	35	67	42	137	151	0.420
Budak Diler et al. [31]	2018	Turkey	Asian	Prostate cancer	PB	PCR-RFLP	62/113	22	21	19	65	59	30	48	35	108	118	0.114
Budak Diler et al. [31]	2018	Turkey	Asian	Bladder cancer	PB	PCR-RFLP	69/156	24	28	17	76	62	50	62	44	162	150	0.011
Burada et al. [32]	2016	Romania	Caucasian	Gastric cancer	HB	TaqMan	108/242	34	46	28	114	102	47	122	73	216	268	0.755
Cao et al. [38]	2016	China	Asian	Colorectal cancer	HB	Illumina	964/891	384	463	117	1231	697	377	399	115	1153	629	0.558
Castano-Rodriguez et al. [33]	2015	Singapore	Asian	Gastric cancer	HB	MassARRAY iPLEX	86/217	28	49	9	105	67	109	81	27	299	135	0.057
Fernandez-Mateos et al. [34]	2017	Spain	Caucasian	Larynx cancer	HB	TaqMan	213/253	58	108	47	224	202	72	130	51	274	232	0.580
Fernandez-Mateos et al. [34]	2017	Spain	Caucasian	Pharynx cancer	HB	TaqMan	165/253	44	81	40	169	161	72	130	51	274	232	0.580
Fernandez-Mateos et al. [34]	2017	Spain	Caucasian	Oral cavity cancer	HB	TaqMan	72/253	18	31	23	67	77	72	130	51	274	232	0.580
Huijbers et al. [35]	2012	Netherlands	Caucasian	Thyroid cancer	PB	-	139/1964	38	69	32	145	133	378	1029	557	1785	2143	0.012
Nicoli et al. [36]	2014	Romania	Caucasian	Colorectal cancer	HB	TaqMan	109/357	14	52	43	80	138	70	179	108	319	395	0.787
Wisetsathorn et al. [37]	2017	Thailand	Asian	HCC	HB	PCR-RFLP	102/131	65	33	4	163	41	55	65	11	175	87	0.175

**Table 2:** The pooled ORs and 95% CIs for the association between *ATG16L1* rs2241880 polymorphisms and cancer susceptibility.

Genetic Model	Association Test			Heterogeneity Test			Test of Publication Bias	
	OR (95% CI)	Z	*p*	χ2	I^2^ (%)	*p*	Egger’s Test *p*	Begg’s Test *p*
AG vs. AA	0.94 (0.74–1.20)	0.48	0.63	33.17	67	0.000	0.425	0.411
GG vs. AA	0.93 (0.72–1.20)	0.55	0.58	22.30	51	0.022	0.726	0.891
AG + GG vs. AA	0.94 (0.74–1.19)	0.53	0.60	35.55	69	0.000	0.523	0.891
GG vs. AG + AA	0.98 (0.81–1.18)	0.25	0.80	17.76	38	0.087	0.677	0.493
AG vs. GG + AA	0.97 (0.80–1.17)	0.36	0.72	27.55	60	0.004	0.321	0.411
G vs. A	0.97 (0.84–1.12)	0.45	0.65	31.99	66	0.001	0.567	0.583

**Table 3:** Stratified analysis of the *ATG16L1*, rs2241880 polymorphism on cancer susceptibility.

Type of Cancer	N	AG vs. AA		GG vs. AA		AG + GG vs. AA		GG vs. AG + AA		AG vs. GG + AA		G vs. A	
		OR (95% CI)	P	OR (95% CI)	P	OR (95% CI)	P	OR (95% CI)	P	OR (95% CI)	P	OR (95% CI)	P
Cancer type													
Digestive tract system	4	1.19 (0.71–1.98)	0.51	1.05 (0.65–1.70)	0.85	1.17 (0.72–1.92)	0.52	1.00 (0.81–1.22)	0.98	1.12 (0.78–1.62)	0.54	1.09 90.84–1.41)	0.51
Colorectal cancer	2	1.16 (0.96–1.40)	0.12	1.32 (0.68–2.55)	0.42	1.21 (0.87–1.67)	0.25	1.06 (0.84–1.34)	0.62	1.10 (0.93–1.30)	0.26	1.16 (0.88–1.54)	0.30
Gastric cancer	2	1.11 (0.25–4.86)	0.89	0.79 (0.33–1.88)	0.59	1.05 (0.27–4.06)	0.95	0.81 (0.53–1.25)	0.35	1.27 (0.43–3.77)	0.67	1.00 (0.52–1.94)	0.99
Head and neck squamous cell carcinoma	3	1.01 (0.76–1.34)	0.94	1.32 (0.94–1.85)	0.11	1.10 (0.84–1.44)	0.49	1.31 (0.99–1.74)	0.06	0.89 (0.70–1.13)	0.35	1.14 (0.97–1.35)	0.12
Ethnicity													
Caucasian	7	0.92 (0.76–1.11)	0.37	1.00 (0.68–1.47)	0.98	0.95 (0.72–1.25)	0.70	1.04 (0.78–1.39)	0.77	0.94 (0.80–1.09)	0.40	1.00 (0.83–1.21)	0.99
Asian	5	0.94 (0.57–1.57)	0.81	0.92 (0.72–1.17)	0.47	0.91 (0.57–1.46)	0.69	0.89 (0.71–1.11)	0.30	1.00 (0.65–1.54)	0.99	0.91 (0.69–1.20)	0.50
